# A rare de novo duplication of chromosome 21q22.12 → q22.3 with other concomitant deletion and duplication of small fragments in 21q associated with Down syndrome: Prenatal diagnosis, molecular cytogenetic characterization

**DOI:** 10.1186/1755-8166-6-11

**Published:** 2013-03-06

**Authors:** Qingwei Qi, Xiya Zhou, Yulin Jiang, Na Hao, Jing Zhou, Liang Zhang

**Affiliations:** 1 Department of Obstetrics & Gynecology, Peking Union Medical College Hospital, Chinese Academy of Medical Science, Peking Union Medical College, Dongdan, Dongcheng District, Beijing, 100730, P. R. China; 2 BioChain (Beijing) Science & Technology Inc., No. 7A, Yongchang North Rd, Business Development Area, Beijing, China

**Keywords:** Array comparative genomic hybridization (aCGH), Fluorescence in situ hybridization (FISH), Sequence-tagged site (STS), Partial duplication, Prenatal diagnosis, Down syndrome

## Abstract

**Background:**

Karyotyping is considered the gold standard for the genome-wide detection of genomic imbalances in prenatal diagnosis, but it has a number of inherent limitations, namely the time required to culture cell and the limited resolution(5 ~ 10 Mb). Although fluorescence in situ hybridization (FISH) can also be used as a rapid prenatal diagnosis for common aneuploidies, it is labor intensive, requires prior knowledge of the regions of interest, and can only be used to diagnose one or a few genomic regions simultaneously. Array comparative genomic hybridization (aCGH) can overcome the resolution, the locus-specific, and the time limitations of the karyotyping and FISH techniques and is currently the most powerful method for detecting chromosomal alterations in pre and postnatal clinical cases. Several investigations have suggested that the aCGH testing should be considered a first-tier test for the diagnosis of cytogenetic aberrations in the fetus.

**Results:**

This study used karyotyping, FISH, sequence-tagged site (STS) analysis and aCGH to diagnose a case of de novo duplication of chromosome 21q22.12 → q22.3 with other concomitant deletion and duplication of small fragments in 21q associated with Down syndrome prenatally.

**Conclusions:**

FISH, aCGH and STS analysis are useful in prenatal investigation of the nature of de novo alterations of small fragments of the chromosome.

## Background

The most common causes of chromosomal abnormalities are autosomal aneuploidy (~75%), polyploidy (~13%), sex chromosomal abnormalities (~8%), and structural imbalances (~4%) [1,2]. Prenatal testing often includes fetal chromosome analysis following amniocentesis or chorionic villus sampling and culturing of the cells obtained by these invasive procedures. G-banding karyotyping of cultured cells has been regarded as the standard method of prenatal cytogenetic diagnosis. The culturing process usually takes several days to a few weeks in order to generate the number of metaphase chromosomes enough for a reportable karyotype report. Karyotyping has proved to be highly reliable for the diagnosis of aneuploidies and larger structural rearrangement (>5-10 Mb) in fetal cells, however, smaller gains or losses of genome cannot be reliably visualized with karyotyping. The supremacy of karyotyping in prenatal diagnosis has been challenged by the introduction of molecular diagnosis methods including interphase fluorescence in situ hybridization (FISH) [3]. FISH is a targeted approach with a higher resolution that allows detection of a duplication or deletion involving single genes or small genomic regions. However, FISH requires prior knowledge of the regions that need to be assessed for any given patient based on a family history or specific clinical findings.

In addition to the common aneuploides, many submicroscopic chromosomal rearrangements that lead to copy-number gains or losses have been shown to cause distinctive and recognizable clinical phenotypes. Genomic microarrays, also termed ‘molecular karyotyping’ [4], are overcoming the resolution, the locus-specific, and the time limitations of the karyotyping and FISH techniques. Within a decade of use, microarrays have been recommended as the first line of assessment of the karyotype, instead of routine banded chromosomes, for children with developmental, intellectual, and physical disabilities [5-8]. Prenatal detection of genomic imbalances using microarrays has been demonstrated over recent years [9-12].

Here we report a case with abnormal ultrasound finding, both the serum screening and cell-free DNA in maternal blood were positive for trisomy 21, the interphase FISH of the amniotic fluid suggested the fetus to be trisomy 21, the karyotyping of the amniotic fluid was 46,XX,21p+, but the array comparative genomic hybridization (aCGH) analysis demonstrated a de novo partial trisomy 21q(21q22.12 → q22.3) and other concomitant small duplications and deletion on 21q. The metaphase FISH of the amniotic cell chromosomes demonstrated that the duplication of 21q(21q22.12 → q22.3) was located on the p arm of one of the chromosome 21.

## Case report

**Figure 1:**
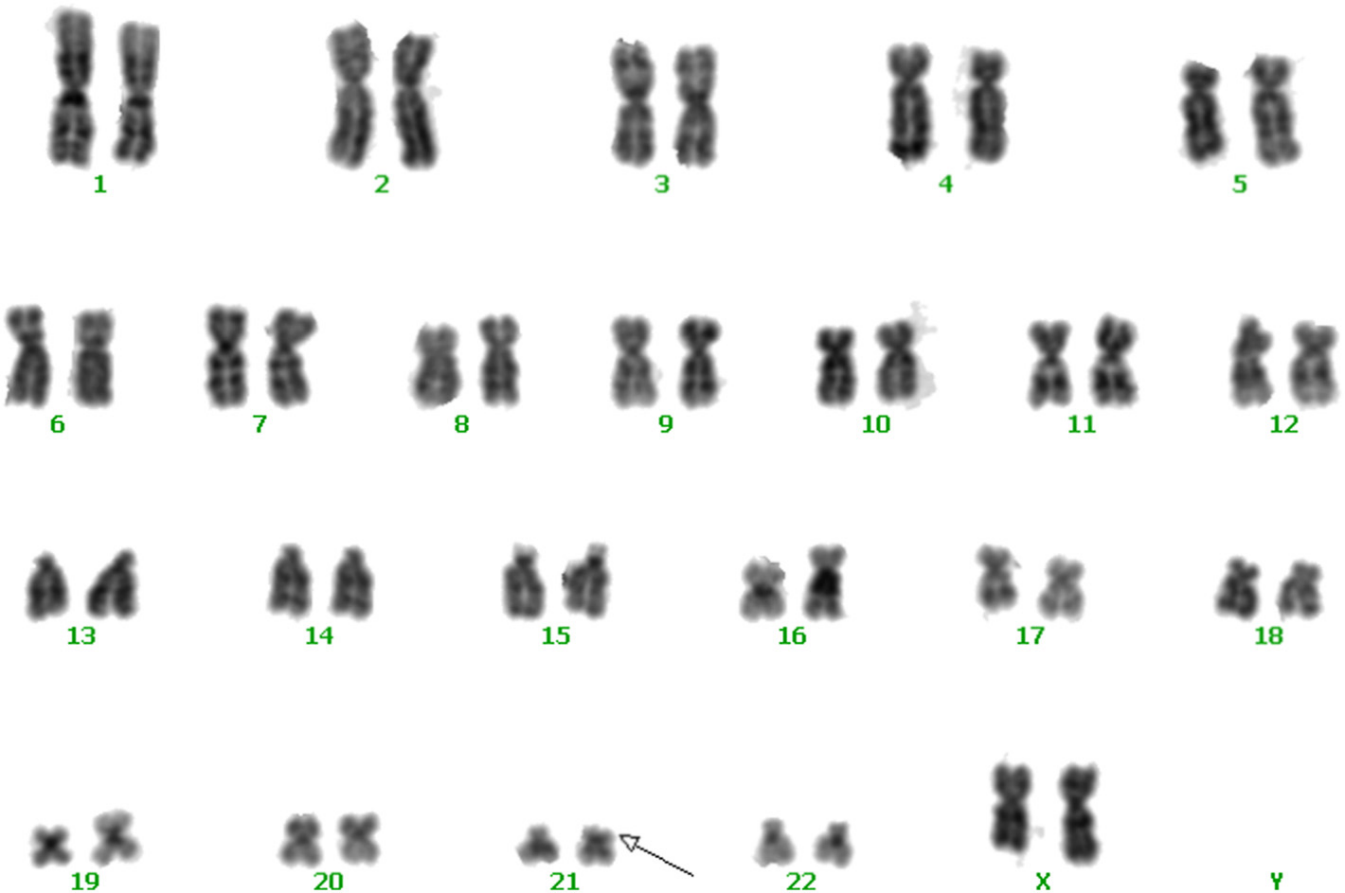
**G**-**banded karyotype shows an unknown duplication on 21p.**

**Figure 2:**
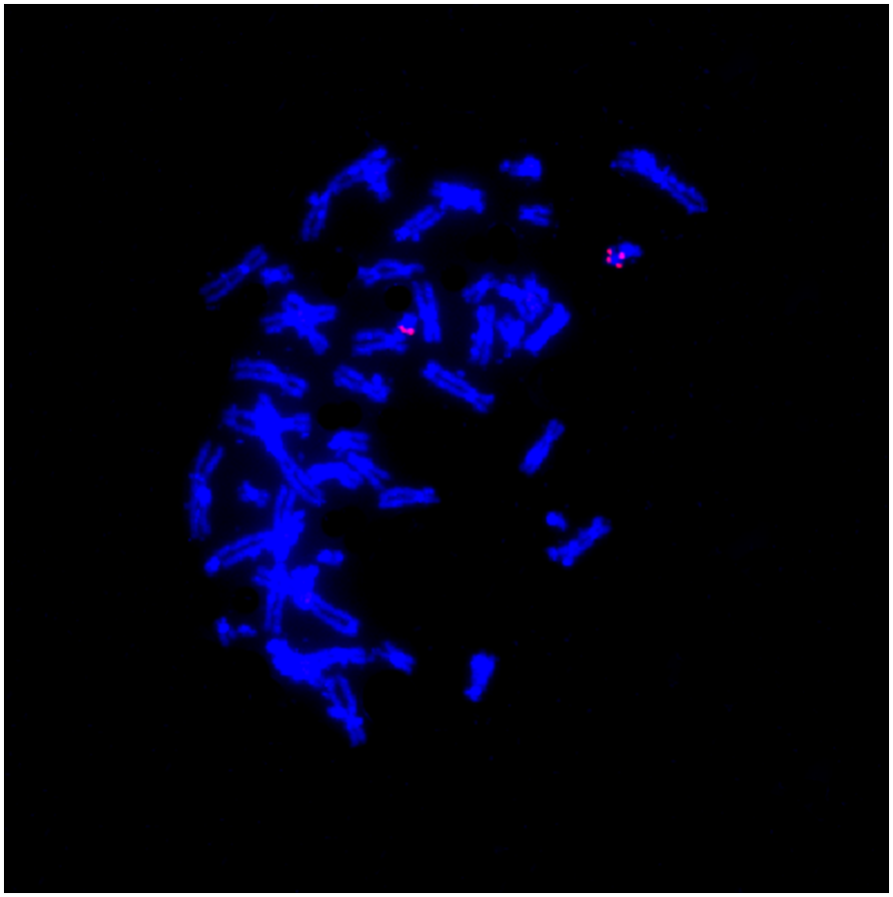
**Fluorescence in situ hybridization analysis using the probe DSCR2:21q22 showed that the segment of the 21p** **+** **was 21q22 in origin.**

**Figure 3:**
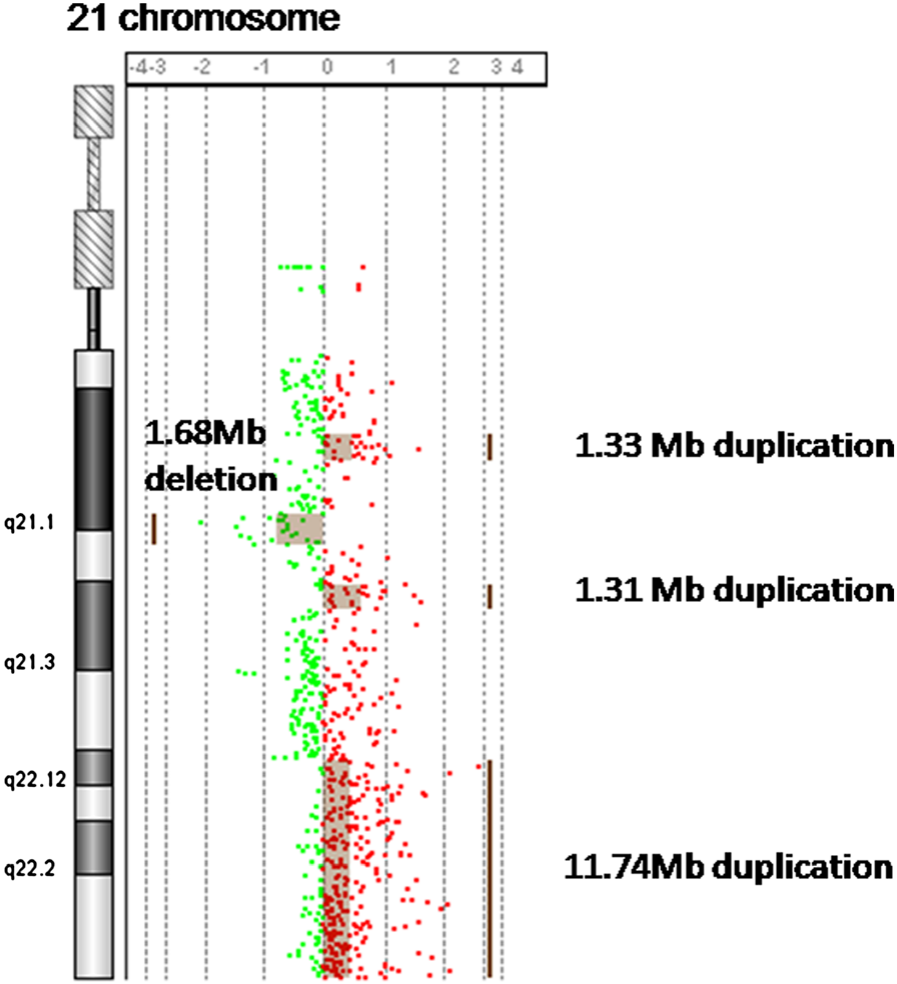
**aCGH results indicated there were three duplications and one deletion in 21q.** The largest duplicated region of 11.74 Mb extending from 21q22.12 to 21q22.3, which should contribute to the fetus clinical characterization of Down syndrome.

**Figure 4:**
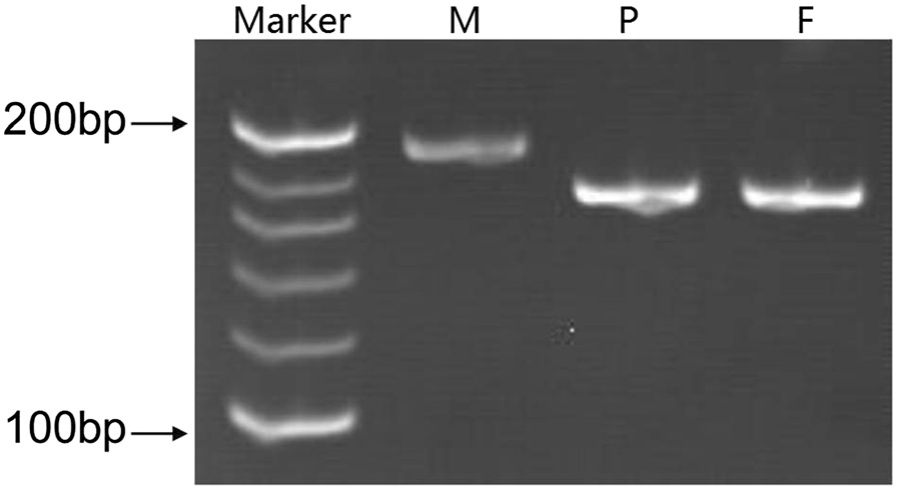
**The Polyacrylamide Gel Electrophoresis (PAGE) results showed that the DNA band of the fetus was the same as father’s, but was lack of the mother’s band.** This indicates that the allele derived from his mother was deleted. M: maternal; P: Paternal; F: Fetus.

A 29-year-old gravida 1, para 0 woman came to our clinic at 15 gestational weeks, the ultrasound showed the nuchal fold of 0.6cm thickness and without other abnormal findings. The maternal serum screening (AFP + free β-hCG + uE3) showed the risk of fetal Down syndrome was 1/110. We performed sequencing analysis of the cell free DNA extracted from the maternal peripheral blood, and the result turned out to be positive for trisomy 21.The amniocentesis was performed at 18 weeks of gestation. The interphase FISH showed three signals of the probe DSCR2:21q22. However, the amniocentesis revealed a karyotyping of 46,XX,21p + (Figure 1). Chromosome preparations of the blood lymphocytes from the parents revealed normal karyotypes. The metaphase FISH analysis with the probe DSCR2:21q22 showed that the segment of the 21p + was 21q22 in origin (Figure 2). The aCGH analysis demonstrated a 11.74 Mb duplication of 21q22.12-q22.3, a 1.31 Mb duplication of 21q21.3, a 1.33 Mb duplication of 21q21.1 and a 1.68 Mb deletion of 21q21.1-21q21.2 (Figure 3). STS (sequence-tagged site) was used to distinguish the parental origin of the 1.68 Mb deletion and the allele derived from the mother was deleted (Figure 4).

**Figure 5:**
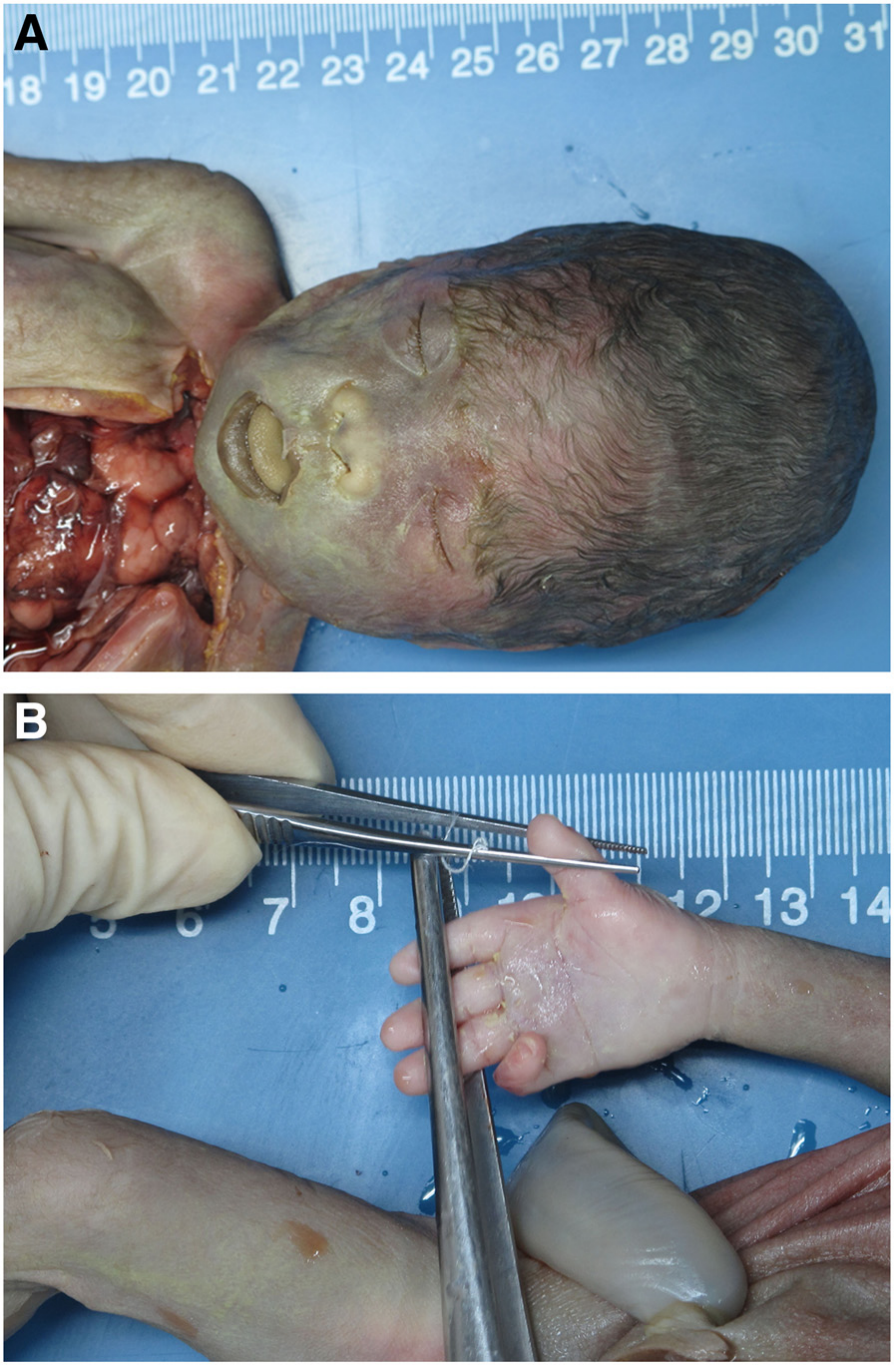
**(A) Facial profile, hypertelorism, a depressed nasal bridge, a protruding tongue and (B) a single crease on the right hand at birth.**

The parents opted to terminate the pregnancy. A malformed female fetus with some characterization of Down syndrome was delivered with a flat facial profile, hypertelorism, a depressed nasal bridge, a protruding tongue, loose folds in posterior neck, and a single crease on the right hand (Figure 5). A formal autopsy was performed by the Department of Pathology of our hospital. Besides of the above phenotypes, the subcutaneous edema was found in the neck and the chest, but no abnormalities of the heart or the other organs were found.

## Discussion

We have presented prenatal molecular cytogenetic characterization of a second trimester fetus with some clinical features of Down syndrome resulting from a partial trisomy 21q due to a de novo duplication of 21q22.12 → q22.3. The aCGH analysis demonstrated that the present case had a duplication of 21q22.12 → 22.3 (chr21:36326031–48067924) (11.74 Mb), a duplication of 21q21.3 (chr21: 26975925–28285899) (1.31 Mb), a duplication of 21q21.1 (chr21: 18867555–20196174) (1.33 Mb) and a deletion of 21q21.1 - q21.2 (chr21: 23127142–24811889) (1.68 Mb). The complex rearrangements in the 21 chromosome in this case don't belong to interstitial inverted duplication with concomitant terminal deletion, which was frequently caused by U-type exchange. Reviewing all the proposed mechanisms, it is still difficult to interpret the mechanism for the complex rearrangement happened on this case. Down syndrome involving a duplication of the Down syndrome critical region (DSCR) accounts for less than 1% of the cases with Down syndrome [13]. DSCR is the critical region on chromosome 21 of which the duplication is responsible for the majority of phenotypic features in Down syndrome.

The duplication of 21q21.3(chr21: 26975925–28285899)(1.31 Mb) encompasses the gene APP (OMIM 104760), which accounts for the phenotype of familial Alzheimer disease 1 and cerebral amyloid angiopathy. We now know that essentially all individuals with Down syndrome develop Alzheimer disease-like pathology by the fourth decade of life. APP (amyloid beta A4 precursor protein) (gene map at 21q21.3) gene is found in the Down syndrome obligate region, and the protein is overexpressed in the adult Down syndrome brain [14,15]. One of the patients characterized, a 65-year old without an additional copy of APP, did not have dementia or indication of amyloid accumulation when assessed by brain imaging, supporting a causative role for APP overexpression in neuropathology in Down syndrome [16]. Overexpression of APP leads to dysfunction of the endocytic system, increased amounts of APP in the Down syndrome brain result in increased amounts of *β*-amyloid (A*β*) and extracellular plaque formation beginning early in life, which is the main driver of Alzheimer Disease-like pathology in the brains of elderly Down syndrome individuals [17,18].

The duplication of 21q21.1(chr21: 18867555-20196174)(1.33 Mb) encompasses the gene ENTK(OMIM 606635). ENTK (enterokinase) is an intestinal enzyme responsible for initiating activation of pancreatic proteolytic proenzymes (trypsin, chymotrypsin and carboxypeptidase A). It is reasonable to deduce that this region has no correlation with the phenotype and pathophysiology of Down syndrome.

**Figure 6:**
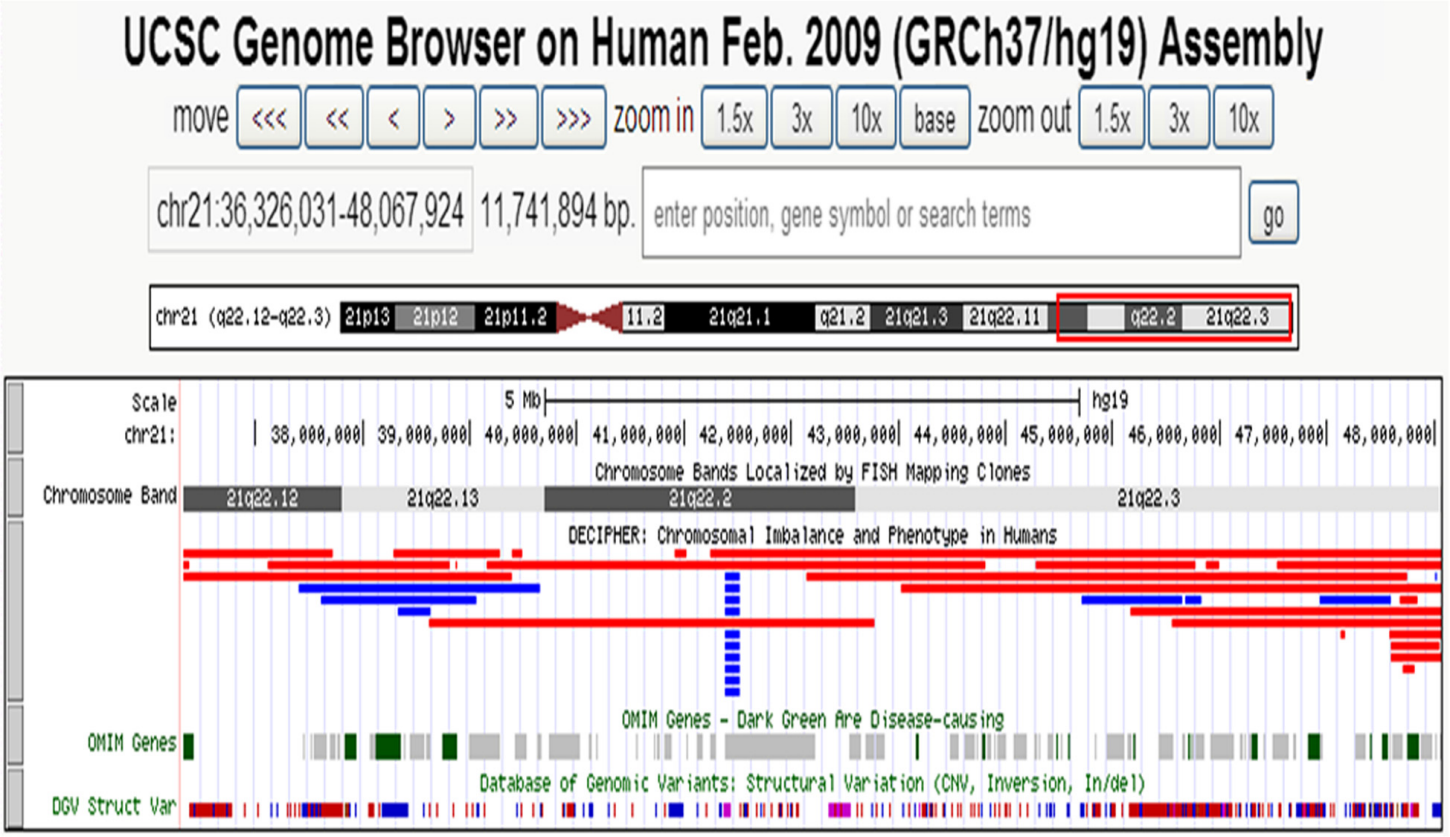
**This is the screenshot from the UCSC genome browser for the fragment of 21q22.12 → 22.3.** For Decipher Database, the entries of clinical cases are in red for deletions (mean log ratio < 0) and in blue for duplications (mean log ratio > 0). For Database of Genome Variants (DGV struct Var), inversions are in purple, whereas InDels are in blue if there is a gain in size relative to the reference, in red if there is a loss in size or in brown if there are reports of both a loss and a gain in size.

The duplication of 21q22.12 → 22.3 (chr21: 36326031–48067924)(11.74 Mb) (Figure 6) encompasses some disease-causing genes, such as DYRK1A (OMIM 600855), ITGB2(OMIM 600065), CLDN14 (OMIM 605608), HLCS (OMIM 609018), COL18A1 (OMIM 120328) and PCNT (OMIM 605925). Among these genes, DYRK1A (OMIM 600855) is associated with the pathophysiology of Down syndrome. DYRK1A (dual-specificity tyrosine phosphorylation-regulated kinase 1A) (gene map at 21q22.1) encodes a member of the dual-specificity tyrosine phosphorylation-regulated kinase family and participates in various cellular processes. It is a highly conserved gene located in the DSCR region [19]. DYRK1A has been suggested to be involved in the abnormal neurogenesis found in Down syndrome [20]. Arron et al., [21] reported that 2 genes, DSCR1 (OMIM 602917) and DYRK1A act synergistically to prevent nuclear occupancy of NFATc(OMIM 600489) transcription factors, which are regulators of vertebrate development. The 1.5-fold increase in dosage of DSCR1 and DYRK1A cooperatively destabilizes a regulatory circuit, leading to reduced NFATc activity and many of the features of Down syndrome. Overexpression of DSCR1 will inhibit calcineurin activity and causes accumulation of hyperphosphorylated tau protein and production of neurofibrillary tangles causing Alzheimer’s disease [22]. Ryoo et al., [23] showed that mice overexpressing human DYRK1A had elevated levels of threonine-phosphorylated tau, which is found in insoluble neurofibrillary tangles in Alzheimer disease brains. DYRK1A phosphorylated tau on threonine and serine residues in vitro. Phosphorylation of tau by DYRK1A reduced the ability of tau to promote microtubule assembly. Ryoo et al., [23] concluded that an extra copy of DYRK1A can contribute to early onset of Alzheimer disease.

As to the deletion of 21q21.1 - q21.2 (chr21: 23127142–24811889) (1.68 Mb), there was no OMIM gene within this region. It is reasonable to deduce that this region has no correlation with the phenotype and pathophysiology of Down syndrome.

To date, at least 39 cases of Down syndrome with pure partial tirsomy 21 have been reported [24,25], but the prenatal diagnosis of pure partial trisomy 21q associated with Down syndrome is quite rare. Lee et al., [26] reported a case of prenatal diagnosis of pure trisomy 21q(21q13 → q22.2) due to an unbalanced cryptic insertion (4;21)(q21;q22.1q22.3) inherited from the carrier father. The fetus had a karyotype of 46,XX,der(4)ins(4;21)(q21;q22.13q22.2)pat. The abnormal prenatal findings included a maternal serum screening Down syndrome risk of 1:17 and a thick nuchal fold. The fetus was delivered with clinical features of Down syndrome. Chen et al., [27] reported a case of prenatal diagnosis and molecular cytogenetic characterization of de novo partial trisomy 21q(21q22.11 → qter) associated with clinical features of Down syndrome. Oligonucleotide-based aCGH demonstrated a 14.8 Mb duplication of distal 21q. The karyotype of the fetus was 46,XX,der(9)t(9;21)(q34.3;q22.11). The abnormal prenatal finding included an abnormal level II ultrasound at 20 weeks of gestation of clinodactyly and hypoplastic midphalanx of the fifth fingers, midface hypoplasia and an intracardiac echogenic focus.

Our case manifested a thickening of nuchal fold of the fetus. The maternal serum screening (AFP + free β-hCG + uE3) showed the risk of fetal DS was 1/110. The following analysis of cell-free DNA in maternal blood turned out to be positive for trisomy 21. FISH analysis of amniotic fluid demonstrated a duplication of 21q22 on the short arm of the chromosome 21. Array-based CGH analysis demonstrated a 11.74 Mb duplication on 21q22.12 → 22.3 (chr21: 36326031–48067924) and a 1.31 Mb duplication on 21q21.3(chr21: 26975925–28285899). These two regions encompass the genes associated with the phenotype and pathopysiology of Down syndrome.

Prenatal studies in ongoing pregnancies using aCGH has been developed quickly in the recent years. Hillman et al., [28] recently performed a meta-analysis of eight studies of array findings in prenatal diagnosis. The meta-analysis found that microarray testing increased the detection of chromosome abnormalities by 2.9% over routine karyotyping. Recently Shaffer et al., [29] reviewed their results of prenatal diagnosis in over 5000 pregnancies with micro-array based comparative genomic hybridization. The overall detection rate of clinically significantly copy number alterations among unbiased, nondemised cases was 5.3%. Detection rate was 6.5% for cases referred with abnormal ultrasounds. The author concluded that the microarray testing should be considered a first-tier test for the diagnosis of cytogenetic aberrations in the fetus. Current ACOG guidelines suggest that karyotypic analysis by aCGH should be considered as an adjunct rather than a replacement for conventional karyotyping [30]. In that opinion paper, they recommended that microarray testing should be limiting to those pregnancies that show abnormal ultrasound findings and that it should be performed in conjunction with a routine banded karyotype. In this case report, aCGH is the useful tool to demonstrate the de novo chromosomal abnormality of the fetus when the ultrasound showed the abnormally thickening of the nuchal fold of the fetus, this also supports the current guideline of ACOG.

In conclusion, aCGH is useful for rapid identification of the genomic imbalance associated with de novo alterations in small fragments of the chromosome, and FISH is also useful in prenatal investigation of the nature of a de novo alterations of the chromosome. The combination of ultrasound and the molecular cytogenetic analysis are essential for the prenatal diagnosis of partial trisomy 21q.

## Materials and methods

### Chromosome analysis

Chromosome analysis using GTG-banding was done according to standard procedures. A total of 20 metaphase cells were analyzed. Karyotypes were described according to the International System for Human Cytogenetic Nomenclature.

### Molecular cytogenetics

#### FISH analysis

FISH using red color probe (GLP 21 probe kit, Beijing GPmedical technologies, Ltd, Beijing, China) was applied according to manufacturer’s instructions. A total of 100 interphase amniotic cells and 20 metaphase spreads were analyzed respectively.

#### cffDNA analysis

For cell-free DNA analysis, 5 ml peripheral blood was drawn into an EDTA containing Vacutainer tube. Within 6 hours of blood collection, plasma was separated and plasma DNA was extracted using the QIAamp Circulating Nucleic Acid kit from Qiagen (Hilden, Germany). The sequencing analysis was conducted at Berry Genomics Co, Ltd., located in Beijing, China. Plasma DNA was used as the input DNA to make a library for sequencing analysis, using a modified ChIP Seq protocol. For chromosome 21 of each sample, the Z scores (cutoff = 3) were calculated to determine if a sample is aneuploidy as reported previously [31].

#### aCGH analysis

Genomic DNA was extracted from 10 ml of amniotic fluid with a commercially available Amniotic Fluid Genomic DNA Extraction Kit (BioChain Institute Inc., Newark, CA) according to the manufacturer's instructions. For each aCGH experiment, 400 ng of genomic DNA and normal female DNA (BioChain Institute) was digested with 10 U Alu I and 10 U Rsa I (Promega, Madison, WI) and differentially labeled with cyanine-5 (cy5) and cyanine-3 (Cy3) fluorescent dyes using a Genomic DNA Enzymatic Labeling Kit (Agilent, Santa Clara, CA). The aCGH analysis was performed using 8 × 60 K commercial arrays (Agilent). This platform contains 60-mer oligonucleotide probes spanning the entire human genome with an overall mean probe spacing of 50 kb. After hybridization, the arrays were scanned using a dual-laser scanner (Agilent) and the images were extracted and analyzed using Feature Extraction software (Agilent) and Workbench genomics software, respectively.

### Confirmation the deletion of 21q21.1 - q21.2 using STS markers

STS (sequence-tagged site) is a short DNA sequence that has a single occurrence in the genome and whose location and base sequence are known. It can easily be used to distinguish individuals and detect microdeletions. Since aCGH result indicated that there was a 1.68 Mb deletion in 21q21.1-21q21.2 between two duplication fragment,which was generally rare to happen, eight STS markers located on the deletion region were selected from UniSTS database [http://www.ncbi.nlm.nih.gov/unists/] for PCR amplification from the parents and amniotic fluid to validate the 1.68 Mb deletion. The Polyacrylamide Gel Electrophoresis (PAGE) results showed that only D21S1409 was informative for deciphering the allelic origin of the deletion. The DNA band of the fetus was the same as father’s, but was lack of the mother’s band. This indicates that the allele derived from his mother was deleted (Figure 4).

## Consent

The DNA research protocol was approved by Ethics Committee in Peking Union Medical College Hospital. Written informed consent was obtained from the patients parent/ guardian for publication of this case report and accompanying images. A copy of the written consent is available for review by the Editor-in-Chief of this journal.

## Abbreviations

aCGH: Array comparative genomic hybridization; FISH: Fluorescence in situ hybridization; cffDNA: Cell-free fetal DNA; STS: Sequence-tagged site; DSCR: Down syndrome critical region; ACOG: American College of Obstetrics and Gynecology; DYRK1A: Dual-specificity tyrosine phosphorylation-regulated kinase 1A; ENTK: Enterokinase; APP: Amyloid beta A4 precursor protein; PAGE: Polyacrylamide Gel Electrophoresis.

## Competing interests

The authors declare that they have no competing interests.

## Authors’ contributions

NH and JZ performed the cytogenetic studies and FISH analysis in the present case. XZ and YJ collected the data relative to this case report. LZ did the aCGH and STS analysis and interpretation. All authors contributed to the finalizing of the manuscript. All authors read and approved the final manuscript.
